# Radar-Based Activity Recognition in Strictly Privacy-Sensitive Settings Through Deep Feature Learning

**DOI:** 10.3390/biomimetics10040243

**Published:** 2025-04-15

**Authors:** Giovanni Diraco, Gabriele Rescio, Alessandro Leone

**Affiliations:** National Research Council of Italy, Institute for Microelectronics and Microsystems, 73100 Lecce, Italy; alessandro.leone@cnr.it

**Keywords:** human activity recognition, FMCW radar, deep feature learning, privacy

## Abstract

Human activity recognition in privacy-sensitive environments, such as bathrooms, presents significant challenges due to the need for non-invasive and anonymous monitoring. Traditional vision-based methods raise privacy concerns, while wearable sensors require user compliance. This study explores a radar-based approach for recognizing the activities of daily living in a bathroom setting, utilizing a BGT60TR13C Xensiv 60 GHz radar, manufactured by Infineon Technologies AG (Munich, Germany, EU), to classify human movements without capturing identifiable biometric features. A dataset was collected from seven volunteers performing ten activities which are part of daily living, including activities unique to bathroom environments, such as face washing, teeth brushing, dressing/undressing, and resting on the toilet seat. Deep learning models based on pre-trained feature extractors combined with bidirectional long short-term memory networks were employed for classification. Among the 16 pre-trained networks evaluated, DenseNet201 achieved the highest overall accuracy (97.02%), followed by ResNet50 (94.57%), with the classification accuracy varying by activity. The results highlight the feasibility of Doppler radar-based human activity recognition in privacy-sensitive settings, demonstrating strong recognition performance for most activities while identifying lying down and getting up as more challenging cases due to their motion similarity. The findings suggest that radar-based human activity recognition is a viable alternative to other more invasive monitoring systems (e.g., camera-based), offering an effective, privacy-preserving solution for smart home and healthcare applications.

## 1. Introduction

The ability to perform activities of daily living (ADLs) is a fundamental indicator of an individual’s functional independence and overall well-being. In elderly individuals, changes in ADL performance can signal the onset of cognitive decline or neurodegenerative diseases, making continuous monitoring an essential aspect of healthcare and assisted living. The early detection of irregularities in movement patterns can provide critical information for caregivers and healthcare professionals, enabling timely intervention to prevent falls, injuries, or complications related to mobility impairment [1].

Various sensor-based approaches have been explored in the literature for ADL detection, ranging from wearable sensors to environmentally embedded sensors. Wearable sensors, such as accelerometers, gyroscopes, and inertial measurement units (IMUs), are commonly used for activity tracking due to their ability to provide high-frequency motion data. However, these devices require users to wear them continuously, which can be uncomfortable and lead to compliance issues, particularly among the elderly. In contrast, non-wearable sensors, including cameras, depth sensors, infrared sensors, and radar systems, allow for unobtrusive activity monitoring without requiring physical attachment to the body. Among these, vision-based systems, such as monocular cameras, stereo cameras, and RGB-D sensors, have demonstrated high accuracy in human activity recognition (HAR). However, these methods raise privacy concerns as they capture detailed images that may contain personally identifiable information [2].

While several studies have attempted to anonymize visual data or develop techniques such as federated learning to ensure that data processing remains within a private network [3], the most effective privacy-preserving approach is to use sensors that inherently protect user anonymity. Radar sensors fulfill this criterion by design. Unlike cameras, radars do not capture explicit visual or biometric details but rather rely on the emission and reflection of radio waves, allowing for precise motion detection without revealing the physical characteristics of the monitored individual. Radar-based HAR is gaining interest due to its non-invasive nature, resilience to environmental conditions, and capability to function in low-visibility settings, such as dark rooms, smoke-filled environments, or occluded spaces [4]. Furthermore, radars are capable of detecting subtle physiological signals, including breathing and heart rate, making them well-suited for applications in healthcare, elderly monitoring, and smart home environments [5].

In this study, we employ a BGT60TR13C Xensiv 60 GHz radar sensor [6] to recognize ADLs performed in a bathroom environment, a highly privacy-sensitive setting where traditional vision-based methods are impractical. The 60 GHz radar operates in the millimeter-wave band, providing higher spatial resolution compared to lower-frequency radars such as those operating at 5.8 GHz or 24 GHz. This improved resolution enhances the system’s ability to distinguish fine-grained movements, which is particularly beneficial for detecting activities such as brushing teeth, face washing, and dressing/undressing. The radar’s low power consumption and miniaturized form factor make it ideal for Internet-of-Things (IoTs)-based continuous monitoring, ensuring efficient and discreet ADL recognition in smart home environments.

This study introduces several key innovations that advance radar-based HAR in bathroom environments. Firstly, it employs a state-of-the-art 60 GHz frequency modulated continuous wave (FMCW) radar sensor equipped with three receiving antennas, enabling the parallel processing of range–Doppler maps to preserve angular information. The sensor’s compact dimensions (19 mm × 12.7 mm) facilitate seamless integration within the bathroom environment, such as placement near a mirror above the sink, ensuring non-intrusive deployment.

Secondly, unlike previous studies that primarily focused on basic ADLs, such as walking, sitting, standing, and lying on the floor (as a proxy for falls or medical distress), this work expands the scope to include more specific bathroom-related activities. These include fall recovery (standing up after a fall), brushing teeth, washing the face, and combing hair, thereby improving the system’s applicability in real-world monitoring scenarios.

Thirdly, to enhance feature learning from radar data, this study systematically investigates 16 pre-trained networks (PTNs), evaluating their ability to extract discriminative representations for activity classification. In addition, real-time feasibility is explicitly assessed by measuring the processing time, providing insights into practical deployment. The most lightweight PTNs among those examined are further validated on a low-power edge computing platform, demonstrating suitability for resource-constrained environments.

Finally, the proposed system is evaluated on a diverse dataset, incorporating both male and female participants with varying physical characteristics and a wide age range, ensuring generalizability across different user profiles. Each participant contributed approximately 83 min of recorded data, significantly exceeding the durations typically found in related studies and providing a robust foundation for model training and evaluation.

### Radar-Based ADL Recognition and Related Work

HAR has been extensively explored across various domains, including healthcare, elderly care, smart homes, and security applications. In healthcare settings, radar technology is increasingly valuable for monitoring vital signs and recognizing daily activities, benefiting from its non-intrusive nature and privacy-preserving characteristics [7]. Similarly, unobtrusive sensing systems such as radar are particularly advantageous in elderly care applications, enabling continuous monitoring without affecting user comfort or privacy [8]. Among critical applications in elderly care, fall detection is particularly significant; radar-based systems leveraging Doppler analysis effectively identify the rapid downward movement characteristic of falls, demonstrating high detection accuracy and reliability [9]. Despite the broad spectrum of radar applications described, the remainder of this section specifically concentrates on research aimed at HAR as it constitutes the core topic of the present work.

Several studies have leveraged handcrafted features due to their interpretability and computational simplicity. Li et al. [10] employed 20 handcrafted Doppler features extracted and subsequently reduced through selection processes. Although their bidirectional long short-term memory (BiLSTM) classifier demonstrated effectiveness, the handcrafted approach necessitates significant domain expertise, making feature extraction cumbersome and context-specific. Similarly, Li et al. [4] extracted 68 handcrafted features from micro-Doppler spectrograms, achieving computational efficiency via hierarchical classification and adaptive thresholding. However, like the earlier work, it faced limitations due to lack of angle information, susceptibility to environmental interference at 5.8GHz, and complex hierarchical implementations.

Recent efforts have transitioned towards learned feature approaches, which typically yield better generalization and less manual effort. Cao et al. [11] advanced the state-of-the-art by combining time–range and time–Doppler maps using a multi-feature attention fusion module and training with VGGNet (VGG13), leveraging learned features to reduce misclassification. Yet, despite superior accuracy, this approach did not address the radar-target angle, which could enhance spatial context in activity recognition.

Other studies have utilized radars equipped with multiple outputs (i.e., receiving antennas), which in principle allow for the capture of angular information. However, in several cases, this angular information was not explicitly incorporated into the processing pipeline. Chen et al. [12] utilized a 77GHz radar with multiple receiving antennas, extracting learned Doppler–time maps via dilated convolutions and multi-head self-attention mechanisms. However, despite having multiple receiving channels capable of capturing angular information, the study averaged signals from all antennas into a single representation, effectively discarding spatial diversity. This prevented the model from leveraging angle-of-arrival features that could enhance classification performance.

Similarly, Kurtoglu et al. [13] employed learned features from range–Doppler maps and micro-Doppler spectrograms using CNN-BiLSTM architectures. Despite high accuracy through multi-representation fusion, their methodology neither separately processed data from multiple receiving antennas nor preserved angular information explicitly, potentially limiting spatial differentiation capabilities. Additionally, their small dataset posed significant constraints on generalizability.

Vandersmissen et al. [14] advanced feature extraction by deploying deep learning models on range–Doppler and micro-Doppler data combined with visual inputs. While achieving high accuracy, particularly with 3D CNN architectures, the need for simultaneous video and radar data collection poses privacy and practical implementation challenges, alongside lacking explicit angular data consideration.

To overcome dataset size limitations and enhance generalization, Saeed et al. [15] utilized extensive publicly available datasets, employing ResNet-based learned features with robust performance in controlled scenarios. However, performance significantly dropped in cross-environmental validations, highlighting environmental sensitivity. Since the specific ResNet variant used in their study is not explicitly stated, the computational feasibility for real-time embedded applications remains uncertain, as deeper ResNet architectures tend to be computationally intensive, whereas shallower variants such as ResNet18 offer more efficient alternatives. Additionally, the publicly available dataset used in their study is based on a radar with a single receiving antenna, meaning that angular information is not captured or incorporated into the feature extraction process.

Huan et al. [16] proposed a hybrid CNN-LSTM attention network that efficiently decouples Doppler and temporal features, reducing network complexity while maintaining high classification accuracy. The attention-based feature refinement further enhances activity recognition by focusing on the most relevant motion patterns. However, while the radar utilized multiple receiving channels, angular information was averaged rather than explicitly preserved, potentially limiting spatial differentiation. Additionally, the dataset, despite having a reasonable number of participants, is constrained by a short total data collection duration of only 1000 s, which may impact model generalization.

Specifically targeting restroom scenarios, Saho et al. [17] combined CNN-learned features with handcrafted acceleration and jerk metrics, achieving excellent fall detection accuracy using Doppler signatures to capture motion comprehensively. However, the radar system employed only a single receiving antenna, preventing the capture of angular information. Additionally, the study focused exclusively on predefined restroom activities, omitting other relevant actions such as washing, brushing teeth, combing hair, and attempting to stand up again after a fall. Furthermore, all participants in the study were male, which limits the generalizability of the model to a more diverse population as gender-related differences in movement patterns may influence classification performance.

Finally, Visser et al. [18] demonstrated effective integration of Doppler, azimuth, and elevation data with convolutional and LSTM architectures to extract spatial-temporal learned features, which provided a cost-effective solution while maintaining competitive accuracy. However, their system exhibited confusion between sitting, standing, and lying on the floor, which was used in the study to simulate a fall. The misclassification may stem from feature extraction based on four convolutional layers, which may not sufficiently differentiate between these critical postures. Additionally, the study considers only five activities—walking, sitting on the toilet, standing up from the toilet, washing hands, and lying on the floor (fall)—while neglecting other essential behaviors such as recovering from a fall, brushing teeth, combing hair, and washing the face. Furthermore, the study does not address real-time system performance or its feasibility for use on low-power edge computing platforms, leaving its suitability for deployment in resource-constrained environments an open question.

Recent studies have demonstrated significant advancements in leveraging deep feature learning (DFL) through pre-trained neural networks for radar-based HAR. Pre-trained CNNs, including DenseNet [19], ResNet [20,21], Inception [21], EfficientNet [22], VGG-16 [20], VGG-19 [23], GoogleNet [24], and MobileNetV2 [19,20], effectively extract discriminative features from radar data representations such as micro-Doppler spectrograms and range–Doppler images, significantly enhancing recognition accuracy and computational efficiency.

The application of these architectures substantially reduces the necessity for extensive radar-specific datasets by leveraging transfer learning. Furthermore, hybrid architectures combining 3D and 2D CNN models, such as EfficientNet in the progressively orthogonally mapped EfficientNet architecture [22], outperform traditional approaches by capturing complex spatio-temporal radar features. Additionally, densely connected networks such as Dop-DenseNet [19] demonstrate superior performance by effectively preserving detailed micro-Doppler information essential for precise gesture and activity recognition. Collectively, these works emphasize the suitability of pre-trained CNN architectures for radar-based HAR, particularly beneficial in scenarios with limited data and computational constraints. However, it should be noted that the cited studies employing DFL through PTNs primarily investigated HAR in general contexts and did not specifically address ADLs within bathroom environments.

Notably, publicly available datasets have facilitated advancements in radar-based HAR research. Shah et al. [25] presented a dataset collected using a C-band FMCW radar operating at 5.8 GHz with 400 MHz bandwidth, involving 48 participants performing six common human activities, including walking, sitting, standing, bending, drinking water, and falling. Similarly, Fioranelli et al. [26] introduced another dataset, employing the same type of radar (C-band FMCW at 5.8 GHz, 400 MHz bandwidth), collected from over 50 participants across nine different environments, covering similar activities. However, both datasets were acquired using radar systems equipped with only one receiving antenna, therefore lacking angular information. Furthermore, activities specifically related to bathroom environments were not included in these datasets, making them unsuitable for the context of the present study.

## 2. Materials and Methods

### 2.1. Radar Sensor

The BGT60TR13C Xensiv device used in this work is a state-of-the-art radar sensor designed for advanced sensing applications, particularly in gesture recognition, presence detection, and motion. Developed by Infineon, this ultra-compact radar module operates within the 60 GHz industrial, scientific, and medical (ISM) band and leverages FMCW radar technology for high-precision measurements in complex environments, providing precise range and velocity measurements through chirped frequency modulation.

The radar transmits continuous wave signals with a linearly modulated frequency, enabling precise distance and velocity measurements through Doppler shifts and frequency differences between the transmitted and reflected signals. The system operates within a frequency range of 57–64 GHz, providing a bandwidth of up to 7 GHz, which enables sub-millimeter range resolution [6].

The relation between distance R and time delay T is governed by the following standard formula:

(1) $$R=\frac{cT}{2},$$

where c = 0.29979 m/ns is the speed of light in vacuum. In an FMCW radar, the time delay is inferred from the frequency difference Δf between the transmitted and received signals, given by the following:

(2) $$∆f=\frac{B\;T}{T_{s}},$$

where B is the bandwidth of the chirp signal and $T_{s}$ is the chirp duration.

The BGT60TR13C features integrated antennas, with one transmitting and three receiving channels. Its L-shaped antenna array enables precise horizontal and vertical angular measurements. The radar chip dimensions are compact, typically 4 mm × 5 mm, making it ideal for space-constrained applications. The radar features a programmable transmit power up to 5 dBm, a receiver sensitivity of −80 dBm, and a sampling rate configurable up to 3 (million samples per second) MSPS. A summary of the main system parameters is provided in Table 1. The system supports a flexible frame structure, where each frame consists of multiple chirps. Each chirp involves a frequency sweep over a duration $T_{c}$. The inter-frame interval $T_{f}$, which determines the update rate $f_{u}$, is software-configurable and is given by the following:

(3) $$f_{u}=\frac{1}{T_{f}}\;\;.$$

The radar’s signal processing chain integrates fast time sampling for range resolution and slow time sampling for velocity estimation. The range resolution ΔR is calculated using the formula:

(4) $$\Delta R=\frac{c}{2B}\;,$$

where c is the speed of light and B is the bandwidth. The velocity resolution Δv is derived from Doppler analysis and expressed as follows:

(5) $${∆}_{v}=\frac{\lambda }{2T_{f}\;N_{c}}\;\;,$$

where λ is the radar wavelength, $T_{f}$ is the inter-frame interval, and $N_{c}$ is the number of chirps per frame.

By combining high precision, low power consumption, and advanced signal processing, the BGT60TR13C Xensiv radar system is highly suitable for applications such as ambient assisted living (AAL), where it can monitor vital signs and presence for elderly care [27]. Its compact design, flexible configuration, and robust performance make it a versatile and reliable solution for modern sensing challenges.

When comparing radar systems, it is essential to consider their operating principles and bandwidths as these directly impact their performance and application suitability. For instance, an FMCW radar operating in the millimeter-wave band (e.g., 60 or 77 GHz) provides high range resolution and precise velocity estimation due to its wide bandwidth and Doppler-based processing. Conversely, a pulsed radar system operating in the lower microwave band (e.g., 2–5 GHz) offers longer detection ranges and better penetration through obstacles, though at the cost of reduced resolution [28]. By analyzing their trade-offs in terms of resolution, range, and application scenarios, it is possible to select the most appropriate radar system for a given task.

A key advantage of the BGT60TR13C is its compact size and low power consumption, making it ideal for IoTs applications and for realizing minimally invasive monitoring sensor nodes to be placed in all living environments. It allows for precise angular tracking, and its low-power spectral density minimizes interference with other systems in the 60 GHz band. However, the high frequency of operation limits its penetration depth, making it less suitable for applications requiring obstacle penetration. The BGT60TR13C, while highly versatile, is more sensitive to Doppler ambiguity at high velocities and requires complex signal processing to mitigate interference and multipath artifacts.

The specifications of this work, which focus on monitoring various activities in a domestic environment (bathroom), align well with the capabilities of the Infineon sensor. Since wall penetration is not required, the key priorities are high accuracy, low power consumption, and the ability to deploy compact, non-intrusive devices within the monitoring environment. Table 1 shows the radar configuration data.

### 2.2. Experimental Setup and Data Collection

#### 2.2.1. Participants and Activities

The study involved seven volunteers, comprising four males and three females, with an average weight of 79 ± 22 kg, an average height of 178 ± 16 cm, and an average age of 40 ± 12 years. The participants performed ten ADLs, which are typically carried out in a bathroom or toilet environment. These activities included:

- Dressing/undressing (DRESS)
- Face washing (FACE)
- Hair combing (HAIR)
- Teeth brushing (TEETH)
- Sitting down (SIT)
- Standing up (STAND)
- Resting while sitting (REST)
- Walking (WALK)
- Lying down (LYD)
- Getting up (GTU)

They were selected based on their relevance to daily self-care routines and their potential to indicate health-related conditions, particularly in geriatric care and fall risk assessment.

The DRESS activity was considered as a single category, encompassing both dressing and undressing. Indeed, from a geriatric perspective, the key factor is whether an individual performs this activity independently and regularly, rather than distinguishing between dressing and undressing phases. The FACE, HAIR, and TEETH activities were performed while standing in front of the sink mirror, simulating real-life scenarios where individuals stand to wash their face, comb their hair, or brush their teeth.

The SIT and STAND activities were treated as separate categories, as their detection plays a crucial role in monitoring both safety and autonomy. From a geriatric perspective, these actions provide insights into functional independence and self-efficacy, such as the duration of sitting in the bathroom or whether the individual successfully transitions between sitting and standing. Considering the typical layout of bathroom environments, where toilets may be positioned either in front of or adjacent to a mirror, SIT/STAND activities were performed in different frontal and lateral orientations with respect to the radar sensor.

Similarly, LYD and GTU were considered separately, given their dual relevance for monitoring safety and assessing independence. LYD in a bathroom environment is an important alarm indicator as it is an uncommon occurrence and may signal illness. The presence of this activity is particularly concerning, given that individuals do not typically lie down on the bathroom floor under normal circumstances. In this study, the LYD activity was simulated by instructing participants to lie down slowly and gently on a rubber mat placed on the bathroom floor, ensuring a controlled and safe execution of the movement. The GTU activity, in turn, provides crucial information for evaluating the urgency of intervention, as it distinguishes between a successful recovery and a prolonged inability to stand up which may necessitate emergency assistance.

#### 2.2.2. Experimental Setup

**Figure 1:**
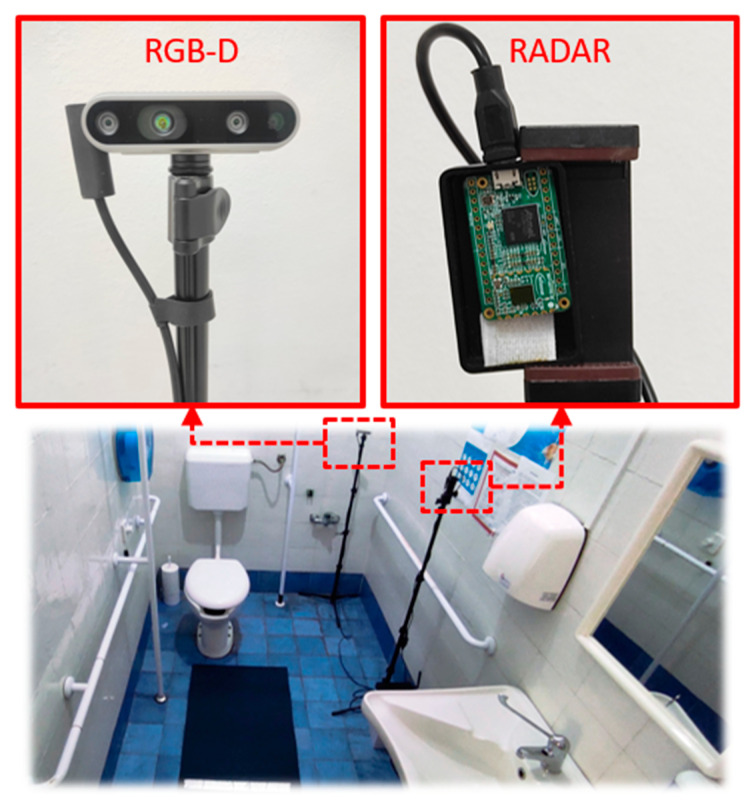
Radar sensor and RGB-D camera mounted on tripods in a bathroom setup including a toilet, sink, and shower.

**Figure 2:**
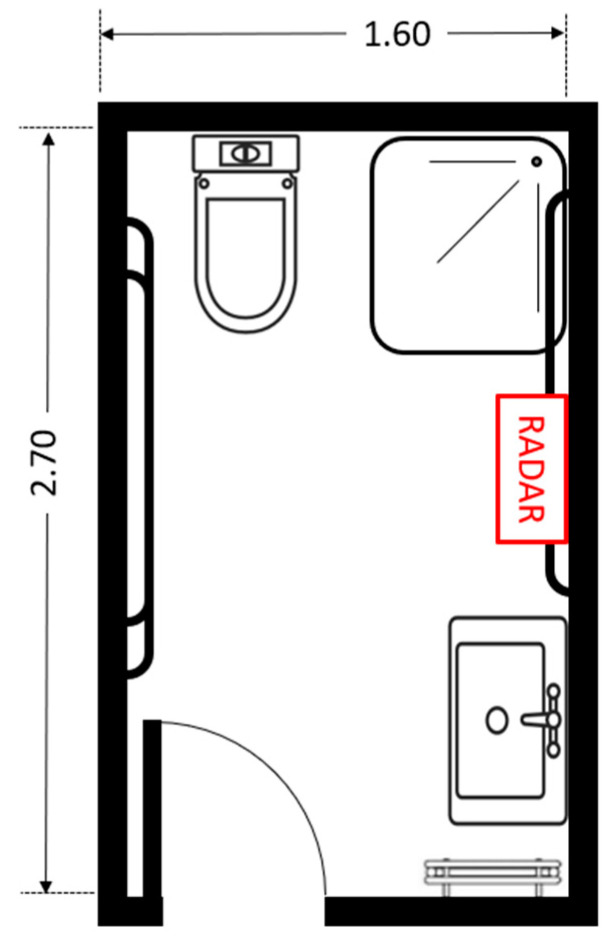
Floor plan of the reference bathroom environment used during data collection.

The radar sensor was positioned at a height of 127 cm from the floor using a tripod. The distance between the sensor and the participant ranged from 1.0 m to 2.5 m. The data collection took place in a controlled bathroom environment, as illustrated in Figure 1. The floor plan of the reference bathroom environment used during data collection is shown in Figure 2.

#### 2.2.3. Ground-Truth Data Annotation

For SIT, STAND, LYD, and GTU activities, ground-truth annotations were generated automatically using a depth camera (Intel^®^ RealSense™ D435i [29]), manufactured by Intel Corporation (Santa Clara, CA, USA). The camera was mounted at the same height as the radar sensor and processed using the MediaPipe library [30], manufactured by Google (Mountain View, CA, USA), to extract body pose information. For all other activities, it was not necessary to use visual recognition algorithms since only one type of activity was performed during the entire session.

#### 2.2.4. Data Collection and Synchronization

For each action, synchronized video and radar sequences were collected using a custom Python-based software (v. 2.7) running on a mini-PC (ThinkCentre M720 Tiny [31], Intel Core™ i5-8400T 2.3 GHz 16GB DDR4-2666), manufactured by Lenovo (Morrisville, NC, USA). The system was capable of sampling RGB-D and Doppler data at 5 frames per second (fps). The compact size of the mini-PC (179 × 183 × 37 mm) facilitated data collection within the bathroom environment. The use of synchronized video sequences enabled the automatic extraction of frames corresponding to each specific action. This was achieved using the MediaPipe library, which analyzed key body landmarks such as the left/left shoulders and left/left hips.

For each participant, an average of 8 min of video and radar data per activity was recorded, except DRESS (11 min per participant), SIT/STAND (19 min per participant), and LYD/GTU (13 min per participant). The total number of frames and the duration of data collection per participant are summarized in Table 2.

#### 2.2.5. Frame Acquisition Strategy

For SIT/STAND and LYD/GTU, a higher number of frames were acquired to facilitate automated distinction between sitting and standing as well as lying down and getting up, leveraging MediaPipe-based processing. Initially, an effort was made to distinguish between dressing and undressing, leading to the collection of extra frames also for the DRESS activity. However, due to the difficulty in automatically differentiating these two phases and the limited geriatric relevance of this distinction, they were ultimately grouped under a single DRESS category.

A sliding window approach was used to segment sequences for analysis, with a window length ranging from 10 to 20 frames whilst using a 1-frame stride for biphasic actions (SIT/STAND and LYD/GTU) and a 2-frame stride for all other actions. The use of two different strides was necessary only to collect an equivalent number of frames for both monophasic and biphasic actions. In a real-time scenario, a uniform stride of 2 frames can be adopted for all activities without affecting performance.

Frames were captured at 5 fps, resulting in window durations between 2 and 4 s, depending on the chosen length. The best results were achieved with windows of 16 samples (using stride 1 or 2), which corresponds to a minimum activity duration of 3.2 s for monophasic actions and 6.4 s for biphasic actions. In real-time operation, setting the system with a stride of 2 frames for all activities ensures that the minimum detectable action duration remains at 6.4 s.

As the number of usable sequences is inversely proportional to the window length, the minimum reference sequence count was set at 500 sequences per activity, corresponding to a 20-frame window.

This comprehensive dataset was designed to support robust deep learning-based activity recognition models, ensuring accurate and reliable classification of daily living activities in privacy-sensitive settings.

### 2.3. Feature Learning and Activity Classification

**Figure 3:**
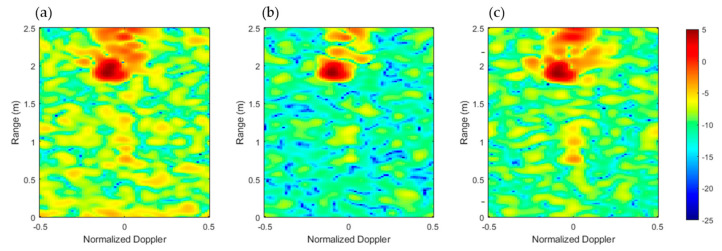
Range–Doppler (dB) frame for antenna no. 1 (**a**), antenna no. 2 (**b**), and antenna no. 3 (**c**), related to WALK activity.

The radar frames, composed of three range–Doppler maps corresponding to three antennas (Figure 3), are represented as three-channel images. These frames are organized into 500 video sequences per action, with a sequence length corresponding to the sliding window size, as discussed in the previous section. To improve the robustness of the training phase, data augmentation is applied, increasing the dataset by 30% (resulting in 650 sequences per action) through reflection transformations on each Doppler channel. It is important to note that data augmentation was applied only during training and no augmentation was used during testing, ensuring a fair and unbiased evaluation of the model’s performance.

In order to identify the optimal augmentation percentage, progressively higher levels of augmentation were tested using the optimized architecture (initially trained without data augmentation) over 10 reduced training epochs. These tests indicated that 30% provided the best trade-off between classification performance and computational efficiency, with further increases yielding only marginal improvements while significantly raising the computational burden. The dataset was then split into 60% for training, while the remaining 40% was equally divided between validation and testing. Specifically, in each run, data from five participants were selected, with three participants (approximately 60% of the data) used for training and the remaining two participants (approximately 40%) equally divided between validation and testing. This procedure was repeated 21 times to account for all possible subsets of five participants drawn from the total of seven. The accuracy values obtained in these runs were then averaged, providing a comprehensive assessment of the model’s performance.

#### 2.3.1. Network Architecture Overview

The proposed deep learning architecture is designed to process range–Doppler sequences and effectively recognize human activities by leveraging spatial and temporal feature representations. The network consists of the following key components:

- Feature Extraction: The input to the network consists of range–Doppler sequences with dimensions (*R* × *C* × 3 × *N*), where *R* and *C* represent the distance and Doppler ranges, 3 accounts for multiple Doppler channels, and *N* corresponds to the window length. A pre-trained deep neural network transforms these sequences into high-dimensional feature representations of size (*M* × *N*), effectively capturing spatial and spectral information. The specific values of *R*, *C*, and *M* for each PTN considered in this study are provided in Table 3, which also details the name of the layer from which the output features are extracted. Additionally, Table 3 includes the number of learnable parameters associated with each PTN, offering insights into the computational complexity of the network and its suitability for real-time applications.
- Temporal Sequence Modeling: The extracted feature sequences are passed through a BiLSTM network with *U* hidden units, which learns temporal dependencies in human activities by processing forward and backward time dependencies inside a sampling window.
- Feature Refinement with Attention: The output of the BiLSTM layer is branched into three parallel fully connected (FC) layers, each with *U* units, followed by rectified linear unit (ReLU) activation functions. These three feature branches serve as inputs to an attention mechanism with three heads, which dynamically assigns higher weights to the most informative temporal features, enhancing activity recognition.
- Final Classification Layers: The attention-weighted features are processed through a dropout layer to prevent overfitting, followed by an FC layer with 10 units (corresponding to the number of activity classes). A SoftMax layer then converts the outputs into a probability distribution over activity classes, and the final classification layer assigns the most probable activity label.

**Figure 4:**
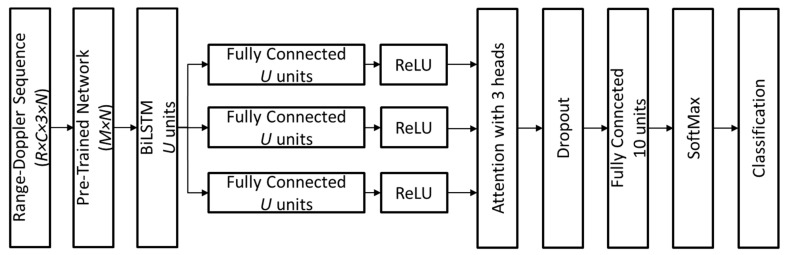
Schematic representation of the deep learning model for radar-based activity classification.

This architecture, represented in Figure 4, ensures that both spatial and temporal dependencies are effectively modeled, leading to robust activity classification in strictly privacy-sensitive settings. The parameters U and N, along with other network hyperparameters, were determined through an optimization process described in detail later in this section.

The 16 PTNs investigated in this study are Googlenet [32], Resnet18 [33], Squeezenet [34], Inceptionv3 [35], Densenet201 [36], Mobilenetv2 [37], Resnet50 [33], Resnet101 [33], Xception [38], Inceptionresnetv2 [39], Shufflenet [40], Nasnetmobile [41], Nasnetlarge [41], Darknet19 [42], Darknet53 [42], and Efficientnetb0 [43].

The pipeline illustrated in Figure 4, which represents the deep learning model for radar-based activity classification, was implemented in MATLAB R2024b (v. 24.1) by MathWorks (Natick, MA, USA) using the deep learning toolbox and parallel computing toolbox. The model was trained and tested on a high-performance workstation equipped with an Intel^®^ (Santa Clara, CA, USA) Core™ i9-10900K CPU @ 3.70 GHz, 64 GB of RAM, and an NVIDIA^®^ (Santa Clara, CA, USA) GeForce RTX™ 4090 GPU (Ada Lovelace) with 24 GB GDDR6X memory and 16,384 NVIDIA CUDA cores. The computational framework leverages GPU acceleration to optimize deep learning operations, ensuring efficient hyperparameter optimization and training for the BiLSTM-based classification model. The parallel computing toolbox was utilized to further enhance data parallelism and optimize performance, particularly during feature extraction using PTNs and sequence processing within the BiLSTM module.

#### 2.3.2. Biomimicry and Sensory Processing: A Parallel to the Animal Sensory System

Radar-based activity recognition shares striking similarities with biological sensory systems, particularly those found in echolocating animals such as bats and dolphins. These animals rely on acoustic signals (sonar) to navigate and identify objects, much like how Doppler radar captures motion-related signals [44].

- Feature Extraction (Sensory Signal Processing): Just as bats’ auditory cortex extracts acoustic features from echoes to discern object shape and movement, the deep learning model extracts feature representations from Doppler spectrograms using pre-trained convolutional networks.
- Temporal Encoding (Sequential Perception in Animals): The sequential nature of movement (e.g., walking, sitting) requires the system to process time-dependent changes, akin to how the nervous system integrates continuous sensory inputs over time to recognize behavioral patterns.
- Attention (Selective Focus in Biological Perception): Animals selectively focus on important stimuli while filtering out noise. Similarly, the attention layer in the proposed model enhances classification by prioritizing the most relevant time steps within the radar sequence.

This biomimetic approach leverages deep learning and computational neuroscience to create an efficient activity recognition framework, maximizing information retrieval while reducing computational complexity.

#### 2.3.3. Long Short-Term Memory Networks for Sequential Data

Recurrent neural networks (RNNs) are a fundamental tool for modeling sequential data, but traditional RNNs suffer from the vanishing gradient problem, which limits their ability to learn long-term dependencies. Long short-term memory (LSTM) networks address this limitation by introducing memory cells that store long-term information, and gates (input, forget, and output gates) that regulate the flow of information, allowing the network to retain or discard past states dynamically [45].

BiLSTM extends LSTM by processing sequences in both forward and backward directions, capturing future as well as past context, similar to how biological sensory systems anticipate and react to environmental changes. This bidirectional property significantly improves recognition accuracy in human activity classification [46].

#### 2.3.4. Attention Mechanism: Inspired by Cognitive Focus

Human perception is not uniform; we instinctively focus on relevant details while ignoring extraneous information. The attention mechanism in deep learning mirrors this cognitive function by assigning higher importance to crucial time steps in the radar sequence and learning to weigh features dynamically, improving model interpretability [47].

In the presented model, the attention layer uses three components—query, key, and value—to refine feature selection. The BiLSTM output is split into three branches, each passing through an FC layer followed by a ReLU activation. These serve as the following:

- Query (Q): The information currently being processed.
- Key (K): The reference feature set.
- Value (V): The weighted feature representation contributing to classification.

By aligning Q, K, and V, the attention mechanism enables context-aware recognition, filtering out irrelevant signals while enhancing motion-specific patterns crucial for accurate classification.

#### 2.3.5. Deep Learning Model Components

The extracted feature vectors undergo sequential processing through a series of neural network layers, beginning with the BiLSTM layer. The number of hidden units (U) in this layer is selected as a multiple of three within the range of 500 to 5000, ensuring compatibility with the three attention heads employed in the architecture. This configuration allows the network to effectively capture and process temporal dependencies in radar-based activity recognition.

Following the BiLSTM layer, the attention mechanism is introduced to dynamically adjust feature weights and emphasize the most relevant temporal patterns. The dropout probability for this layer is set between 0 and 0.3, a range carefully chosen to prevent overfitting while preserving the network’s generalization capability.

After the attention processing, the model incorporates a dropout layer with a probability ranging from 0.1 to 0.6, followed by an FC layer, which maps the learned feature representations to class labels. This step refines the extracted features and prepares them for final classification.

The classification process concludes with a SoftMax layer, which transforms the outputs into a probability distribution over the possible activity classes. The final classification layer then assigns the most probable label to each input sequence based on the SoftMax output.

To optimize the learning process, the network is trained using the Adam optimizer, with mini-batch sizes selected between 8 and 64 to balance computational efficiency and model performance. Gradient clipping is applied with threshold values chosen between 0.5 and 5, ensuring stable convergence by preventing excessive gradient updates. Finally, Bayesian optimization is employed for hyperparameter tuning, systematically refining the model configuration to achieve optimal performance with minimal computational overhead.

The proposed network architecture is trained using a singular loss function applied across all networks considered in this study. Specifically, we employ the categorical cross-entropy (CCE) loss, which is the standard choice for multi-class classification tasks. Given that the output of the network consists of probability distributions over 10 activity classes, the CCE loss is used to measure the discrepancy between the predicted probabilities and the ground truth labels. The loss function is defined as follows:

(6) $$L_{CCE}=-\sum_{i=1}^{S}y_{i}log({\hat{y}}_{i})\;\;,$$

where *S* = 10 is the number of activity classes, $y_{i}$ is the true one-hot encoded label for class i, and ${\hat{y}}_{i}$ is the predicted probability for class i.

This unified loss function ensures consistent backward propagation, allowing gradient updates to propagate seamlessly from the classification layer back through the attention module, BiLSTM, and the feature extraction network, enabling end-to-end training.

#### 2.3.6. Bayesian Optimization for Hyperparameter Selection

Hyperparameter tuning in deep learning presents a computational challenge, as evaluating all possible parameter combinations through an exhaustive search can be resource intensive. Bayesian optimization provides an alternative approach by leveraging a probabilistic model to estimate the objective function and systematically selecting promising hyperparameter configurations. This method balances exploration, which seeks to investigate under-explored regions of the parameter space, with exploitation, which refines previously evaluated promising configurations [48].

At the core of Bayesian optimization is a Gaussian process (GP) model, which approximates the relationship between hyperparameter values and model performance. The GP model not only estimates the objective function but also quantifies the uncertainty associated with its predictions, guiding the selection of subsequent hyperparameter evaluations [49].

To determine which hyperparameter set to evaluate next, the algorithm employs an acquisition function. This function prioritizes points in the parameter space that are expected to improve the model’s performance, using criteria such as the expected improvement or upper confidence bound. Instead of relying on random sampling, this approach directs the search toward configurations that are likely to yield beneficial results while avoiding unnecessary evaluations.

The optimization process involves iteratively refining hyperparameters based on the GP model’s predictions. The process continues until a predefined stopping criterion is met, such as reaching a fixed number of evaluations or achieving convergence in the objective function. By structuring the search in this manner, Bayesian optimization aims to identify suitable hyperparameters with fewer iterations compared to traditional search methods, potentially reducing computational costs while maintaining classification effectiveness.

## 3. Results

The classification performance achieved using each of the 16 PTNs in conjunction with the BiLSTM-based network is presented in Table 4 and Table 5. Table 4 reports the average classification accuracy both with and without (w/o) data augmentation, while Table 4 provides accuracy values for each activity. The introduction of data augmentation yielded improvements ranging from approximately 4% to 11%, with greater benefits observed for networks featuring a larger number of parameters. The highest overall classification accuracy was obtained using DenseNet201, which achieved 97.02% accuracy. The activities LYD (lying down) and GTU (getting up) exhibited slightly lower accuracy compared to other actions, indicating a greater challenge in distinguishing between these specific activities.

Among the tested networks, ResNet50 ranked second in terms of overall classification performance. Although its average accuracy was lower than that of DenseNet201, it provided the highest accuracy for the LYD activity. Both DenseNet201 and ResNet50 demonstrated optimal performance when trained with a sliding window length of 16 samples, suggesting that this window size effectively captures the necessary temporal dynamics for activity recognition. To further analyze the classification outcomes of the DenseNet201-based architecture, a confusion matrix is provided in Table 6. This matrix highlights the classification reliability for each activity, with misclassifications primarily occurring between LYD and GTU, as expected from the results in Table 5. The overall accuracy for other activities remained consistently high, confirming the effectiveness of feature extraction and sequential modeling for radar-based activity recognition.

The optimized hyperparameters used in the architectures based on DenseNet201 and ResNet50, obtained through Bayesian optimization, are detailed in Table 7. The parameters include number of hidden units (NumHiddenUnits), dropout rate (DropRate), mini-batch size (MiniBatchSize), initial learning rate (InitialLearnRate), gradient threshold (GradientThreshold), dropout probability of the attention layer (DropoutProbability), and number of attention heads (NumHeads). The optimization process identified different configurations for each network, reflecting their distinct architecture and feature extraction properties.

Overall, the results indicate that DenseNet201 and ResNet50 offer the most effective feature representations for Doppler radar-based activity recognition. While other networks such as InceptionV3 and ResNet101 also achieved competitive results, the combination of feature extraction capability and temporal modeling was most successful in DenseNet201-based classification. The observed variations in performance across activities suggest that certain motions, particularly LYD and GTU, require further refinement in feature representation and sequence modeling to improve classification robustness.

The computational efficiency of the classification models was assessed in terms of total objective function evaluation time and average inference time per test window. The DenseNet201-based model required a total optimization time of 86,349.38 s, with an average inference time of 0.83 milliseconds per window. In comparison, the ResNet50-based model demonstrated a shorter total optimization time of 62,760 s but exhibited a higher average inference time of 1.58 milliseconds per window.

These results indicate that while ResNet50 required less overall optimization time, its per-window inference time was nearly double that of DenseNet201. This suggests that DenseNet201, despite its increased computational demand during training and optimization, provides a more efficient real-time inference capability, which is a critical factor in deploying radar-based activity recognition systems in real-world applications. The difference in inference times can be attributed to variations in network architecture, feature extraction complexity, and internal processing mechanisms, which influence computational efficiency during deployment.

Furthermore, to assess the feasibility of deployment on edge computing nodes, the classification models based on the lighter PTNs, including EfficientNetB0, GoogleNet, MobileNetV2, ResNet18, ShuffleNet, and SqueezeNet, were evaluated on an NVIDIA Jetson Nano equipped with an NVIDIA Maxwell GPU (128 CUDA cores) and 4 GB of RAM. The accuracy performances obtained were identical to those presented in Table 4, confirming the effectiveness of these models even in resource-constrained environments. Additionally, the average inference times per window, reported in Table 8, demonstrate that the selected PTNs achieve inference times compatible with real-time execution, making them well-suited for practical edge-based deployment.

To provide a comprehensive overview of the radar-based activity recognition process, the range–Doppler maps corresponding to all ten activities considered in this study are presented in Appendix A. These visualizations offer additional insight into the Doppler signatures and range characteristics associated with different actions, complementing the quantitative analysis provided in this section.

## 4. Discussion

This study focuses on the recognition of ADLs in privacy-sensitive environments such as bathrooms. The necessity to ensure privacy precludes the use of sensors capable of capturing detailed intensity images (e.g., monocular/stereo cameras) or even depth maps (e.g., time-of-flight or RGB-D cameras). Instead, a radar-based approach is employed, leveraging the BGT60TR13C Xensiv 60 GHz radar for activity detection. While an RGB-D camera was used for ground-truth annotation during data collection, all computational methodologies were tested exclusively on radar data to ensure adherence to privacy-preserving principles.

The 60 GHz radar operates within the millimeter-wave band, which provides significantly higher spatial resolution compared to lower-frequency alternatives such as 24 GHz or 5 GHz radars. This high frequency enables precise detection of fine-grained human movements, facilitating the recognition of activities such as standing up, sitting down, walking, and brushing teeth by distinguishing even small variations in motion. The ability to accurately differentiate between subtle motion transitions is particularly critical in elderly monitoring and smart home applications, where identifying independent or assisted movement patterns can provide valuable insights into an individual’s physical condition.

The BGT60TR13C Xensiv radar is optimized for advanced signal processing, reducing interference and noise to improve activity identification, even in multi-user scenarios. This capability is particularly relevant for applications in assisted living environments, where multiple individuals may be monitored simultaneously. Additionally, its compact size and low power consumption make it an ideal candidate for IoTs-based solutions, enabling long-term continuous monitoring with minimal energy consumption. Unlike optical-based technologies, 60 GHz radars operate independently of lighting conditions, making them effective in diverse ambient lighting scenarios, including low-light or completely dark environments.

The choice of frequency and radar type plays a crucial role in determining the system’s applicability. FMCW radars, such as the BGT60TR13C, are particularly well-suited for short-range monitoring, offering high range resolution and Doppler-based motion analysis. In contrast, pulsed radars, which operate at lower frequencies, provide improved obstacle penetration but at the cost of reduced resolution and are more cumbersome. For ADL recognition, where distinguishing fine movements is essential, the high-resolution capability of FMCW radar makes it the preferred choice.

The results in Table 4 demonstrate that feature extraction using different PTNs produces varying classification performances across ADLs. Generally, higher accuracy is observed for activities such STAND, REST, FACE, TEETH, WALK, and HAIR. These activities typically involve distinct motion patterns, making them easier to recognize using radar Doppler spectrograms.

Among the evaluated PTNs, DenseNet201 and ResNet50 achieved the highest classification accuracy. DenseNet201 exhibited the best overall performance, with an accuracy of 97.02%, followed by ResNet50 (94.57%). The high accuracy of these networks can be attributed to their deep feature extraction capabilities, which enhance the model’s ability to differentiate between similar actions. However, DenseNet201 requires higher computational resources during training, whereas ResNet50 demonstrates a more balanced trade-off between training complexity and inference efficiency. Notably, DenseNet201 exhibited lower inference time (0.83 ms per window) compared to ResNet50 (1.58 ms per window), making it a more suitable candidate for real-time applications in low-power embedded systems commonly used in IoTs-based ADL monitoring. Furthermore, to evaluate the feasibility of edge deployment, the selected lightweight PTNs were tested on an NVIDIA Jetson Nano. The measured average inference times per window ranged from 35 ms to 62 ms, demonstrating computational efficiency suitable for real-time execution in resource-constrained environments.

The classification accuracy per activity shows that the lowest-performing categories are LYD and GTU, as evident in Table 4 and the confusion matrix in Table 5. The confusion matrix reveals that these activities are often misclassified as each other, suggesting that the model tends to interpret LYD and GTU as phases of the same activity rather than distinct actions. However, misclassifications with other activities remain minimal, indicating that feature extraction and sequential modeling are effective for most ADLs.

In terms of hyperparameter optimization, Bayesian optimization was employed to fine-tune the DenseNet201 and ResNet50 architectures. As detailed in Table 6, the optimal configurations differed between the two networks. DenseNet201 was configured with 1026 hidden units, a dropout rate of 0.457, and an initial learning rate of 2.7599 × 10^−5^, whereas ResNet50 required 4802 hidden units and a lower dropout rate (0.208). These differences suggest that ResNet50 requires a larger number of neurons to compensate for its shallower architecture compared to DenseNet201’s densely connected layers.

To contextualize the obtained results, a comparison is provided in Table 9 with related works on radar-based HAR. In addition, for greater clarity and relevance, Table 10 presents a focused comparison limited to the specific set of activities examined in this study. It is important to clarify that the comparative performance results reported in this study were obtained using different datasets. A direct comparison of the same dataset—either publicly available or the one collected in this study—was not feasible for several reasons.

Regarding open-source datasets, they typically employ radars equipped with a single receiving antenna, rendering them incompatible with our proposed framework, which explicitly leverages angular processing to effectively recognize more complex activities. Conversely, most existing methods considered in our comparative analysis do not incorporate angular information, except for the work by Visser et al. [18], and thus cannot be effectively evaluated using our angular-dependent data.

To the best of our knowledge, Visser et al. [18] is the only previous study employing three separate range–Doppler maps derived from three antennas, making it compatible with our methodology and suitable for testing with our dataset. However, their method relies on training an ad hoc convolutional neural network specifically designed for feature extraction, in contrast to our transfer learning-based approach utilizing pre-trained networks. As future research, we plan to investigate the employment of custom-designed neural architectures and systematically compare them with pre-trained networks, thereby providing comprehensive insights into the relative advantages and disadvantages of each approach.

While several previous studies have investigated ADL recognition using radar, only the works by Saho et al. [17] and Visser et al. [18] have addressed a subset of activities related to the bathroom environment. Nonetheless, neither of these studies has considered the full range of bathroom-specific activities included in the present work. According to the comparative results shown in Table 10, this study achieves competitive or superior accuracy across activities common to these previous works.

In particular, our method outperforms both prior approaches in the recognition of critical activities such as LYD, which may indicate emergency scenarios. Moreover, our approach also achieves higher accuracy in DRESS. Similarly, our approach maintains comparably high accuracy for SIT and STAND activities, closely matching or slightly exceeding prior results. Additionally, activities such as TEETH, FACE, HAIR, REST, and GTU (recovering from a fall) were uniquely evaluated in our study, highlighting the broader coverage and enhanced practical relevance of the proposed approach.

Studies such as Li et al. [10], Cao et al. [11], and Chen et al. [12] report high classification accuracy for activities like walking (95–98%), sitting down (78–92%), and standing up (74–100%). The performance achieved in this study for these actions aligns well with previous findings, with WALK reaching 97.87% and STAND achieving 100% accuracy.

Certain studies, including Vandersmissen et al. [14] and Kurtoglu et al. [13], evaluated more diverse and dynamic activities, such as drumming, ironing, and folding laundry, which were not included in this dataset. Nonetheless, their reported accuracy for standing up (100%) and sitting down (100%) suggests that these actions are well-suited for radar-based recognition, particularly when using high-resolution frequency bands like 77 GHz.

A notable difference arises when comparing our work with Saeed et al. [15], who reported 100% accuracy for walking and standing activities. However, their dataset consisted of only 1026 micro-Doppler signatures in total, with approximately 170 samples per activity, making their findings less generalizable. Additionally, the dataset description lacks sufficient detail, limiting the ability to assess its diversity and real-world applicability.

Overall, this study’s results align with state-of-the-art benchmarks, demonstrating that radar-based ADL recognition is feasible and effective using deep learning models. The high classification accuracy observed in most activities suggests that Doppler spectrogram-based analysis is a promising alternative to other monitoring systems (e.g., camera-based), especially in strictly privacy-sensitive settings like bathrooms.

### Limitations and Considerations in Multi-Person Environments

Radar sensors typically face significant challenges in distinguishing or tracking an individual’s movements when multiple people are present within the sensing range. For this reason, radar technology is naturally applied in scenarios where there is a reasonable certainty that only one person is within range, such as a bathroom or in monitoring situations where an older adult spends most of their time in a specific location (e.g., a chair or a bed). In a previous study by the authors [5], the presence of additional individuals was investigated in the context of vital sign detection. Those results showed that, if other subjects remain more than 0.5 m away from the monitored person and do not obstruct the line of sight between the sensor and the target, the accuracy loss is approximately 2.61% for heart rate and 4.88% for respiration.

In this work, we specifically focus on the case of a single person in the bathroom environment because the monitoring of bathroom-related ADLs is primarily intended for assessing an older adult’s independence. The presence of additional individuals would imply that the older adult is not autonomous, thus defeating the purpose of evaluating self-sufficiency. Nevertheless, based on our previous experiments, it is reasonable to assume that if other individuals are located more than one meter away and do not stand between the radar sensor and the monitored subject, the detection performance does not deteriorate significantly. Furthermore, given that the radar range is limited to a maximum of 2.5 m and the radar frequency employed has minimal penetration through walls, the presence of other moving subjects in different areas of the home is unlikely to interfere with the monitoring in the designated bathroom environment.

## 5. Conclusions

This study demonstrates the feasibility of radar-based HAR in privacy-sensitive environments, specifically focusing on ADLs performed in a bathroom setting. By leveraging a BGT60TR13C Xensiv 60 GHz radar, this work evaluates the ability of deep learning models to classify subtle human movements in a privacy-preserving way (i.e., without capturing biometric details).

A dataset was collected from seven volunteers performing ten ADLs, including face washing, brushing teeth, dressing/undressing, sitting, and standing up, activities that have not been systematically studied in previous radar based HAR research. The classification framework incorporated pre-trained feature extractors and a BiLSTM network with an attention mechanism, allowing for effective modeling of sequential radar data. Among the 16 PTNs tested, DenseNet201 achieved the highest classification accuracy (97.02%), followed by ResNet50 (94.57%). While most activities were recognized with high reliability, LYD (lying down) and GTU (getting up) exhibited a slightly lower classification accuracy, likely due to their similar motion patterns and overlapping Doppler characteristics.

Future research should focus on enhancing the differentiation between LYD and GTU by incorporating contextual information and temporal dependency modeling, which could improve recognition accuracy for actions with overlapping motion characteristics. Additionally, expanding the study to include a larger pool of volunteers will be useful for increasing dataset diversity and improving generalizability. The real-world deployment of radar-based ADL recognition systems will also be crucial for evaluating their robustness under varying spatial constraints, occlusions, and user-specific variations. Further advancements will involve the inclusion of additional ADLs, such as body washing and entering/exiting the bathtub, to establish a more comprehensive and realistic radar-based activity recognition framework suitable for healthcare and smart home applications. Moreover, future research will investigate feature learning using custom-designed neural networks and systematically compare their performance with the pre-trained networks presented in this study, thereby providing insights into the relative advantages and limitations of each approach.

## Figures and Tables

**Table 1:** Configurations of the BGT60TR13C device.

Parameter	Configuration
Start frequency	60.75 GHz
Sweep bandwidth	2 GHz
Frame rate	20 Hz
Samples per chirp	64
Sample chirp per frame	128
Doppler resolution	0.156 Hz
Range resolution	0.075 m
Transmit output power	5 dBm

**Table 2:** Data collection.

Action	Tot. Frames	Frames/Part.	Min./Part.
DRESS	23,044	3292	11
FACE	17,360	2480	8
HAIR	17,080	2440	8
SIT/STAND	39,264	5609	19
REST	17,080	2440	8
TEETH	17,320	2474	8
WALK	17,276	2468	8
LYD/GTU	26,528	3790	13

**Table 3:** PTN configurations and complexity.

PTN	Input Size (*R* × C × 3)	Output Layer Name	Output Size (*M*)	Learnabels (10^6^)
Darknet19	256 × 256 × 3	avg1	1000	20.8
Darknet53	256 × 256 × 3	avg1	1024	41.6
Densenet201	224 × 224 × 3	avg_pool	1920	20
Efficientnetb0	224 × 224 × 3	efficientnet-b0|model|head| global_average_pooling2d|GlobAvgPool	1280	5.3
Googlenet	224 × 224 × 3	pool5-7x7_s1	1024	6.9
Inceptionresnetv2	299 × 299 × 3	avg_pool	1536	55.8
Inceptionv3	299 × 299 × 3	avg_pool	2048	23.8
Mobilenetv2	224 × 224 × 3	global_average_pooling2d_1	1280	3.5
Nasnetlarge	331 × 331 × 3	global_average_pooling2d_2	4032	88.9
Nasnetmobile	224 × 224 × 3	global_average_pooling2d_1	1056	5.3
Resnet101	224 × 224 × 3	pool5	2048	44.6
Resnet18	224 × 224 × 3	pool5	512	11.6
Resnet50	224 × 224 × 3	avg_pool	2048	25.5
Shufflenet	224 × 224 × 3	node_200	544	1.4
Squeezenet	227 × 227 × 3	pool10	1000	1.2
Xception	299 × 299 × 3	avg_pool	2048	22.9

**Table 4:** Average classification accuracy achieved with PTNs used as feature extractors, with and without (w/o) data augmentation (DA).

PTN	Accuracy (%) with DA	Accuracy (%) w/o DA
Densenet201	97.02	91.89
Resnet50	94.57	89.31
Inceptionv3	93.70	88.73
Resnet101	92.78	86.33
Nasnetmobile	92.41	88.64
Mobilenetv2	91.67	87.12
Nasnetlarge	91.60	82.57
Shufflenet	90.43	86.38
Xception	90.37	85.23
Inceptionresnetv2	90.21	84.35
Googlenet	90.11	86.21
Darknet53	90.00	85.36
Efficientnetb0	89.26	85.12
Resnet18	88.89	84.40
Darknet19	83.15	79.13
Squeezenet	79.57	76.39

**Table 5:** Classification accuracy (%) for each activity and PTN.

PTN	DRESS	FACE	HAIR	SIT	STAND	REST	TEETH	WALK	LYD	GTU
Densenet201	100.00	98.94	100.00	97.87	100.00	98.94	98.94	97.87	89.36	88.30
Resnet50	89.36	100.00	97.87	94.68	100.00	100.00	100.00	94.68	92.55	76.60
Inceptionv3	81.48	98.15	100.00	96.30	100.00	100.00	92.59	100.00	83.33	85.19
Resnet101	94.44	98.15	98.15	98.15	98.15	98.15	92.59	96.30	64.81	88.89
Nasnetmobile	88.89	100.00	96.30	96.30	94.44	98.15	92.59	92.59	88.89	75.93
Mobilenetv2	87.04	100.00	77.78	94.44	98.15	100.00	100.00	90.74	81.48	87.04
Nasnetlarge	91.49	91.49	93.62	94.68	100.00	100.00	98.94	94.68	69.15	81.91
Shufflenet	91.49	98.94	96.81	87.23	100.00	97.87	97.87	90.43	85.11	58.51
Xception	74.07	98.15	88.89	94.44	100.00	100.00	96.30	100.00	83.33	68.52
Inceptionresnetv2	79.79	96.81	92.55	91.49	100.00	100.00	98.94	91.49	75.53	75.53
Googlenet	86.17	97.87	98.94	94.68	98.94	79.79	93.62	91.49	75.53	84.04
Darknet53	100.00	100.00	98.15	88.89	100.00	100.00	98.15	85.19	83.33	46.30
Efficientnetb0	85.19	88.89	98.15	87.04	100.00	100.00	100.00	96.30	79.63	57.41
Resnet18	88.89	100.00	94.44	90.74	100.00	100.00	83.33	92.59	81.48	57.41
Darknet19	62.96	85.19	96.30	92.59	90.74	100.00	90.74	85.19	81.48	46.30
Squeezenet	59.57	100.00	70.21	86.17	89.36	93.62	93.62	79.79	61.70	61.70

**Table 6:** Confusion matrix in the case of the PTN Densenet201.

	DRESS	FACE	HAIR	SIT	STAND	REST	TEETH	WALK	LYD	GTU
DRESS	100	0	0	0	0	0	0	0	0	0
FACE	0	98.936	1.0638	0	0	0	0	0	0	0
HAIR	0	0	100	0	0	0	0	0	0	0
SIT	0	0	2.1277	97.872	0	0	0	0	0	0
STAND	0	0	0	0	100	0	0	0	0	0
REST	0	0	0	0	1.0638	98.936	0	0	0	0
TEETH	0	0	1.0638	0	0	0	98.936	0	0	0
WALK	1.0638	0	0	1.0638	0	0	0	97.872	0	0
LYD	1.0638	0	0	1.0638	0	0	0	0	89.362	8.5106
GTU	0	0	0	1.0638	1.0638	0	0	0	9.5745	88.298

**Table 7:** Parameters of Densenet201 and Resnet50 based architectures obtained by Bayesian optimization.

Parameters	Densenet201	Resnet50
NumHiddenUnits	1026	4802
DropRate	0.45718	0.20802
MiniBatchSize	30	15
InitialLearnRate	2.7599 × 10^−5^	2.0308 × 10^−5^
GradientThreshold	4.4117	3.4083
DropoutProbability	0.0073692	0.0049733
NumHeads	4	4

**Table 8:** Average inference time per window on NVIDIA Jetson Nano of selected PTNs.

PTN	Inference Time (ms)
Mobilenetv2	53.13
Shufflenet	45.16
Googlenet	55.42
Efficientnetb0	53.67
Resnet18	61.91
Squeezenet	34.65

**Table 9:** Comparison with related works.

Related Work	Activity (Accuracy %)	Dataset
Cao et al. [11]	Walking (99.56%), sitting down (92.44%), standing up (98.67%), picking up an object (95.11%), drinking (100%), and falling (100%).	Publicly available [25]; 48 participants; FMCW 5.8 GHz radar (1TX-1RX antennas).
Chen et al. [12]	Walking (95,16%), hold in place (93.49%), sitting on the couch (87.55%), rising from the couch (88.56%), drinking (91.51%), falling (94.3%), standing up from the ground (91.98%), and picking up items (93.86%).	Authors collected; 5 participants (4 males, 1 female, 21–25 years); FMCW radar at 77 GHz (1TX-4RX antennas).
Huan et al. [16]	Walking (98%), running (99%), standing up after squatting down (92%), bending (95%), and turning (99%).	Authors collected; 10 participants (7 males, 3 females); FMCW radar at 77 GHz (1TX-4RX antennas).
Kurtoglu et al. [13]	Resting (83.2%), walking (98.8%), sitting (96.3%), standing up (92.8%), folding laundry (97.1%), and ironing (98.2%).	Authors collected; 4 participants; FMCW radar at 77 GHz (1TX-2RX antennas).
Li et al. [10]	Walking (95.6%), sitting on a chair (78.8%), standing up (79.2%), bending to pick up an object (86.3%), drinking water (85.3%), and falling (91.6%).	Authors collected; 16 participants (15 males, 1 female); FMCW 5.8 GHz radar.
Li et al. [4]	Walking (100%), sitting (95.2%), standing (94.2%), picking up an object (84.2%), drinking (86.5%), and falling (96.5%).	Publicly available dataset [26]; from 4 to 20 participants (21–98 years); FMCW radar at 5.8 GHz (1TX-1RX antennas).
Saeed et al. [15]	Bending (94%), drinking (87%), falling (100%), sitting (95%), standing (100%), and walking (100%).	Publicly available dataset [26]; from 4 to 20 participants (21–98 years); FMCW radar at 5.8 GHz (1TX-1RX antennas).
Saho et al. [17]	Opening the toilet lid (93%), pulling down the pants (77%), sitting (100%), taking the toilet paper (90%), standing (100%), pulling up the pants (92%), closing the toilet lid (100%), and falling (100%)	Authors collected; 21 participants (all male, aged 22.4 ± 1.1 years, height: 173.8 ± 5.1 cm); CW radar at 24 GHz (1TX-1RX antennas).
Vandersmissen et al. [14]	Drumming (91.3%), shaking (98%), swiping left (97.5%), swiping right (94.3%), thumb up (80.2%), thumb down (82.9%), entering room (99.5%), leaving room (98.5%), sitting down (100%), standing up (100%), clothe (89.7%), and unclothe (88.8%).	Authors collected; 9 participants; FMCW radar at 77 GHz (1TX-1RX antennas).
Visser et al. [18]	Toilet sit (88.4%), Toilet stand (92.6%), Wash hands (98.2%), Walking (94%), and Fall/Lying Down (83%)	Authors collected; 5 participants; FMCW radar at 60 GHz (1TX-3RX antennas).

**Table 10:** Comparison with related works (restricted to the ADLs considered in this study).

Activity	[4]	[10]	[11]	[12]	[13]	[14]	[15]	[16]	[17]	[18]	Our
DRESS	-	-	-	-	-	90	-	-	92	-	**100**
FACE	-	-	-	-	-	-	-	-	-	-	**99**
HAIR	-	-	-	-	-	-	-	-	-	-	**100**
SIT	95	79	92	86	96	**100**	95	-	**100**	88	98
STAND	94	79	99	89	93	**100**	**100**	-	**100**	93	**100**
REST	-	-	-	-	83.20	-	-	-	-	-	**99**
TEETH	-	-	-	-	-	-	-	-	-	-	**99**
WALK	**100**	96	**100**	95	99	-	**100**	98		94	98
LYD	-	-	-	-	-	-	**100**	-	-	83	89
GTU	-	-	-	**92**	-	-	-	-	-	-	88

**Figure A1:**
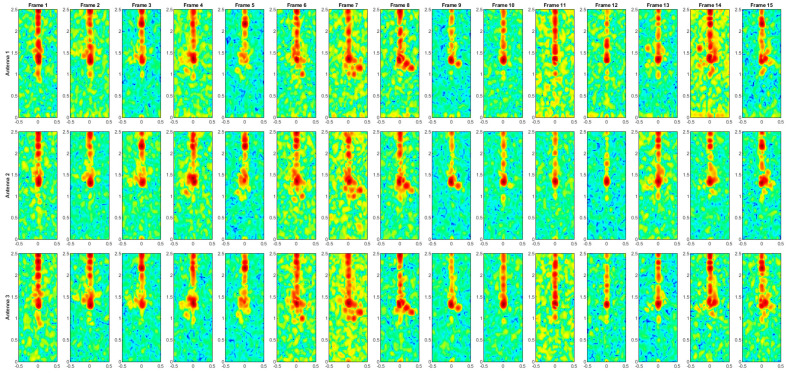
Range–Doppler maps of the activity DRESS.

**Figure A2:**
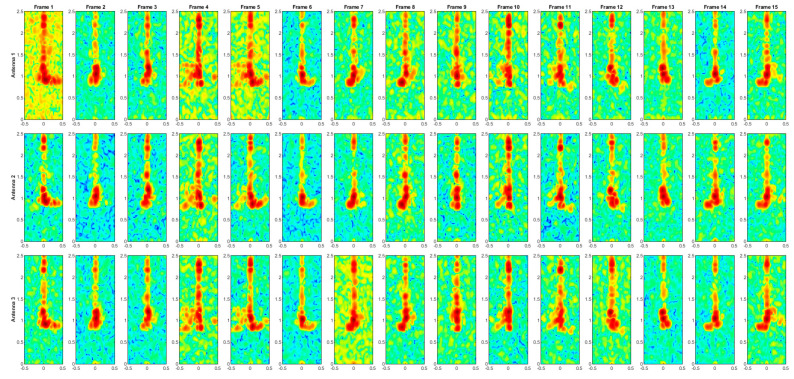
Range–Doppler maps of the activity FACE.

**Figure A3:**
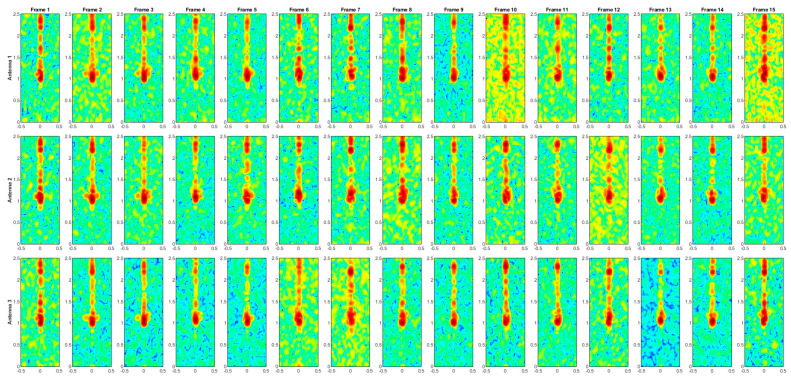
Range–Doppler maps of the activity HAIR.

**Figure A4:**
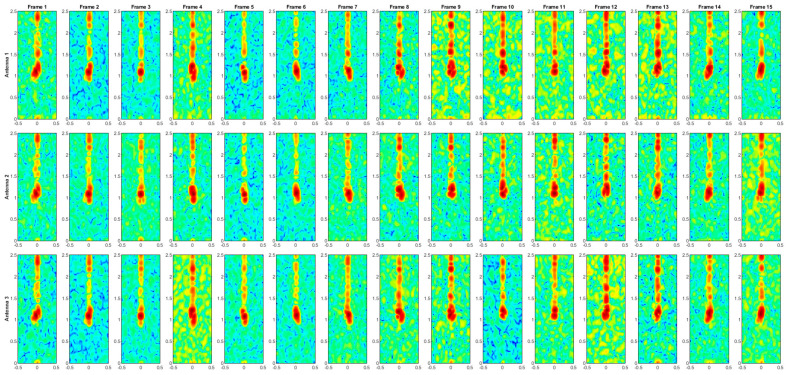
Range–Doppler maps of the activity TEETH.

**Figure A5:**
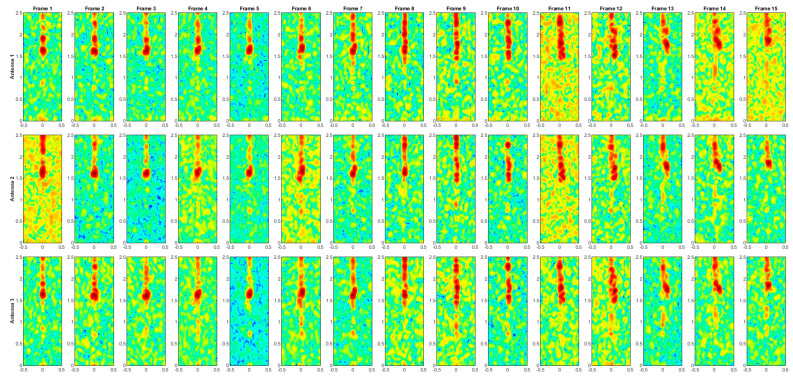
Range–Doppler maps of the activity SIT.

**Figure A6:**
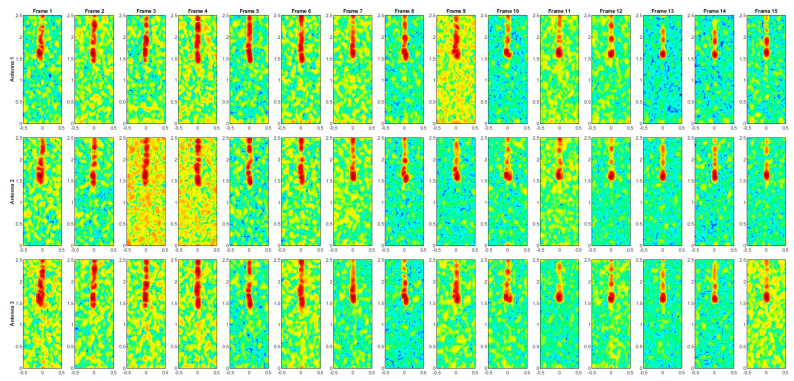
Range–Doppler maps of the activity STAND.

**Figure A7:**
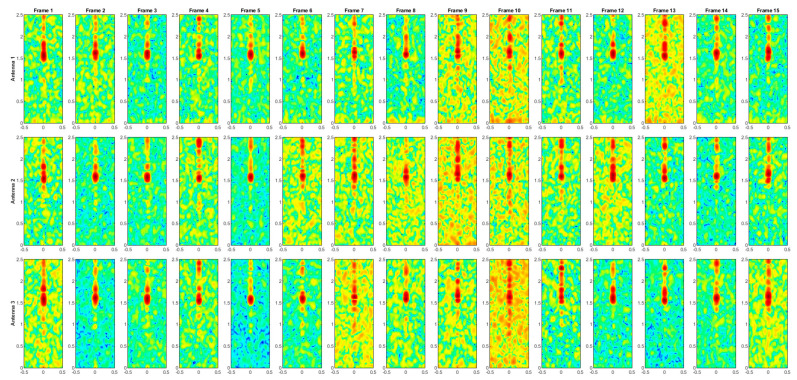
Range–Doppler maps of the activity REST.

**Figure A8:**
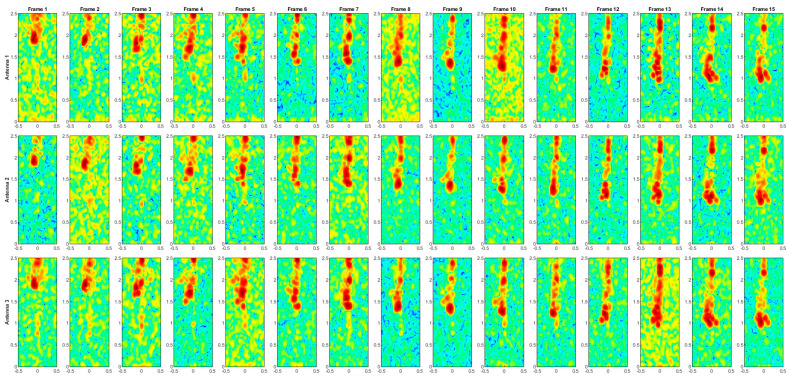
Range–Doppler maps of the activity WALK.

**Figure A9:**
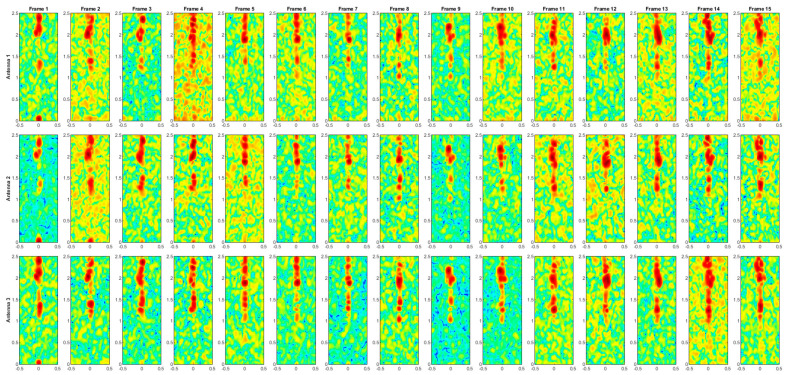
Range–Doppler maps of the activity LYD.

**Figure A10:**
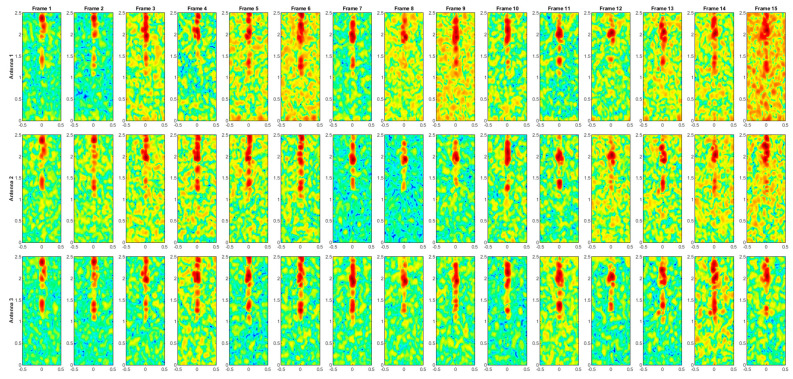
Range–Doppler maps of the activity GTU.

## Data Availability

Data are unavailable due to privacy restrictions.

## Abbreviations

The following abbreviations are used in this manuscript:

AAL	Ambient Assisted Living
ADL	Activity of Daily Living
BiLSTM	Bidirectional Long Short-Term Memory
CCE	Categorical Cross-Entropy
DFL	Deep Feature Learning
DRESS	Dressing/Undressing Activity
FACE	Face Washing Activity
FC	Fully Connected
FMCW	Frequency Modulated Continuous Wave
Fps	Frames per second
GP	Gaussian Process
GTU	Getting Up Activity
HAIR	Hair Combing Activity
HAR	Human Activity Recognition
IMU	Inertial Measurement Unit
IoTs	Internet-of-Things
ISM	Industrial, Scientific, and Medical
LSTM	Long Short-Term Memory
LYD	Lying Down Activity
MFAFN	Multi-Domain Feature Attention Fusion Network
MSPS	Million Samples Per Second
PTN	Pre-Trained Network
ReLU	Rectified Linear Unit
REST	Resting While Sitting Activity
RNN	Recurrent Neural Network
SIT	Sitting Down Activity
STAND	Standing Up Activity
TEETH	Teeth Brushing Activity
WALK	Walking Activity

## Appendix A

To provide a comprehensive visualization of the radar-based activity recognition process, this appendix presents the range–Doppler maps corresponding to all ten activities considered in this study. These representations are valuable for analyzing the discriminative motion patterns captured by the radar system for each action and for understanding the variability of Doppler signatures and range characteristics associated with different activities.

The range–Doppler maps for each activity are presented in Figure A1, Figure A2, Figure A3, Figure A4, Figure A5, Figure A6, Figure A7, Figure A8, Figure A9 and Figure A10. Each sequence consists of 15 frames, as shorter sequences may not sufficiently capture the variability of movement expressed during an action, while longer sequences would be more challenging to represent graphically. The x-axis represents the normalized Doppler, while the y-axis corresponds to the range in meters, as shown in Figure 3. However, axis labels have been omitted in the presented maps to optimize space and improve visual clarity.
